# The Prognostic Importance of Prostate-Specific Antigen in Monitoring Patients Undergoing Maximum Androgen Blockage for Metastatic Prostate Cancer

**DOI:** 10.1100/tsw.2005.19

**Published:** 2005-01-28

**Authors:** Ahmet Kiper, Orhan Yiğitbasi, Abdurrahim Imamoglu, Can Tuygun, Celaleddin Turan

**Affiliations:** Ankara SSK Hospital, 1 Department of Urology, Ankara, Turkey

**Keywords:** prostate cancer, prognosis, prostate specific antigen, metastasis, hormonal treatment

## Abstract

The changes in serum prostate-specific antigen (PSA) concentrations can be used as a prognostic factor in patients undergoing maximum androgen blockage for metastatic prostate cancer. A total of 149 patients followed in our department were classified into 4 groups on the basis of PSA changes: group 1, those with normalisation of PSA levels within the first 3 months; group 2, those with normalisation of PSA between months 3 and 6; group 3, those with a decrease in PSA, but not reaching normal range; group 4, those with no decrease. The gleason scores and the number of bone metastases were also compared between the groups. Again time to progression in patients with Gleason scores 5-7 (grade 2) and over 7 (grade 3) whose PSA levels decreased between first and 3rd months (mean 21.2 months) were significantly longer than the patients with same gleason scores whose PSA levels decreased between 3rd and 6th months (mean 13.4 months) (p < 0.001). The decrease in PSA level is more important than gleason scores in determining the time to progression. Early normalisation of PSA delays the time to progression and when combined with gleason scores, PSA is an important prognostic factor in predicting the success of the therapy.

## INTRODUCTION

Due to substantial variability in the clinical course of metastatic prostate cancer, a number of prognostic factors have been evaluated with respect to their roles in determining the treatment strategy and ability to predict the response to therapy. Among the most extensively studied prognostic factors are age, pain and performance scores, gleason score, pretreatment hemoglobin levels, sedimentation, testosterone levels, and pretreatment PSA levels and the change in PSA levels during treatment. Although PSA is a tumour marker used for the diagnosis of prostate cancer, the value of pretreatment PSA values as a prognostic factor is controversial. Several studies have shown that pretreatment PSA levels are more useful in the diagnosis of the disease and monitorisation of the treatment rather than their use as a prognostic factor[1,2]. Therefore, the importance of the changes in PSA level has been studied in the monitorisation of residual disease after radical prostatectomy, recurrences, response to hormonal therapy, and time to progression.

The impact of the change in PSA levels during treatment on progression and survival in patients with metastatic prostate cancer undergoing maximum androgen blockage was explored retrospectively in this study.

## MATERIALS AND METHODS

This study retrospectively evaluated 149 patients diagnosed with metastatic prostate cancer in our department between 1988 and 2002. The diagnosis was based on rectal examination, serum PSA level, biopsy, and pathological findings. Abdominal CT and/or bone scintigraphy were used in all cases in the search of metastases. All patients received maximum androgen blockage. Patients without concerns for potency underwent bilateral orchiectomy and GnRH analogues (gosereline or leuprolide) were preferred for the other patients. Additionally, maximum androgen blockage was performed with cyproteron acetade 100 mg/daily, flutamide 750 mg/daily, or bicutulamide 50 mg/daily (Table 1).

**TABLE 1**
**The Distrubution of Patients Due to Maximal Androgen Blockage Treatment Protocol**

| Methods of MAB | No. of Patients |
|---|---|
| Cyproteron acetate + bilateral orchiectomy | 41 |
| Cyproteron acetate + leuprolide | 5 |
| Cyproteron acetate + gosereline | 5 |
| Flutamide + bilateral orchiectomy | 25 |
| Bicutulamide + bilateral orchiectomy | 21 |
| Bicutulamide + goserelin | 32 |
| Bicutulamide + leuprolide | 20 |

Each patient was followed up monthly until serum PSA levels returned to normal (0–4 ng/ml) and every 3 months thereafter. During follow-up visits, pain and performance scores, serum PSA, and alkaline phosphatase levels were evaluated routinely; also, further tests were performed on the basis of symptomatology. Patients without a normalisation of PSA levels during the first 6 months of monitorisation were requested to have more frequent clinical visits afterwards. PSA rise on two different occasions after the lowest PSA levels had been recorded was considered the start of the process of progression. We determined pathological findings in prostate carcinoma as gleason scores 2–4 (grade 1), gleason scores 4–7 (grade 2), and over 7 (grade 3).

Patients with normalisation of PSA levels during the first 6 months in addition to those with a rise in PSA levels equal to greater than 50% of the upper limit of normal (<4 ng/ml) were requested to attend clinical visits every 2–4 weeks. Gradually increasing levels of PSA on at least three different occasions were considered as the start of progression, even if pain and performance scores were normal. Patients were classified into four groups on the basis of their follow-up PSA levels. The first and second groups consisted of patients with normalisation of PSA levels (0–4 ng/ml) within the first 3 months of the therapy and patients with normalisation between 3–6 months of therapy, respectively. The third group consisted of patients with a decline in PSA levels that did not reach the normal range. Patients with minimal decrease or increase in PSA levels were considered hormone resistant and constituted the fourth group. For the statistical analyses, t test and Fisher's exact test were used.

## RESULTS

Patient characteristics are shown in Table 2. The first group with normalisation of PSA levels within 1–3 months of therapy consisted of 77 patients (51.5%). The average time to progression was 23.3 months (8–60 months) and the average survival was 37.3 months (12–96 months) in these patients (Table 3); 15 of these patients (19.5%) had grade 1 disease in whom the average time to progression was 32.4 months and the average survival was 50.2 months. The corresponding values for 34 patients (44.2%) with grade 2 disease and 28 patients (36.3%) with grade 3 disease were 22.6 and 36.3 months, and 19.8 and 32.5 months, respectively (Table 3). A significant difference with respect to time to progression and survival was observed between patients with grade 1 disease and patients with grade 2 and 3 disease ($p < 0.02$). There were 53 patients (69%) in group 1 who had less than 6 bone metastases, with an average time to progression of 27 months and survival of 44.1 months. The average time to progression and survival were 15.6 and 25.3 months, respectively, in the remaining 24 patients (31%). Again, there was a significant difference between these two groups with regard to time to progression and survival ($p < 0.01$) (Table 3).

**TABLE 2**
**The Patient Characteristics**

| Characteristic | No. of Patients |
|---|---|
| Age | |
| 45-55 | 18 (12%) |
| 56-65 | 53 (35.5%) |
| 66-75 | 70 (47%) |
| >75 | 8 (5.5%) |
| PSA | |
| 0-4 | 8 (5.4%) |
| 4-20 | 18 (12.1%) |
| >20 | 123 (82.5%) |
| Grade | |
| 1 | 17 (11.4%) |
| 2 | 69 (46.3%) |
| 3 | 63 (42.3%) |
| No. of bone metastases | |
| <6 | 78 (52.4%) |
| 6 or >6 | 71 (47.6%) |

There was a total of 21 patients (14%) in group 2. The average time to progression was 16.9 months (6–36) and survival was 31.3 months (9–72 months) (Table 4). Only two patients had grade 1 disease and the time to progression was 39 months and survival was 62 months in these two cases. The corresponding values for 13 patients with grade 2 disease were 16.6 and 29 months and 6 patients with grade 3 disease were 10.3 and 23 months, respectively (Table 4). Due to the scarce number of patients with grade 1 disease in this group, only grade 2 and 3 patients were compared,

**TABLE 3**
**The Patient Characteristics in Group 1**

| Characteristic | No. of Patients | Progression Time (Months) | Survival Time (Months) |
|---|---|---|---|
| Grade | | | |
| 1 | 15 | 32.4 | 50.2 |
| 2 | 34 | 22.6 | 36.3 |
| 3 | 28 | 19.8 | 32.5 |
| No. of bone metastases | | | |
| <6 | 53 | 27.0 | 44.1 |
| 6 or >6 | 24 | 15.6 | 25.3 |
| Total | 77 | 23.3 | 37.3 |

**TABLE 4**
**The Patient Characteristics in Group 2**

| Characteristic | No. of Patients | Progression Time (Months) | Survival Time (Months) |
|---|---|---|---|
| Grade | | | |
| 1 | 2 | 39 | 62 |
| 2 | 13 | 16.6 | 29 |
| 3 | 6 | 10.3 | 23 |
| No. of bone metastases | | | |
| <6 | 14 | 18.2 | 35.1 |
| 6 or >6 | 7 | 14.4 | 24.3 |
| Total | 21 | 16.9 | 31.3 |

and a significant difference in favour of grade 2 was detected ($p < 0.01$). There were 14 patients with less than six bone metastases with an average time to progression and survival of 18.2 and 35.1 months, respectively. The remaining seven patients had more than six bone metastases and their average time to progression and survival were 14.4 and 24.3 months, respectively (Table 4). No significant differences were noted between group 1 and group 2 ($p < 0.2$).

There was a total of 23 patients in group 3 whose PSA values did not decrease to normal values despite antiandrogen treatment. Mean progression time is 8.45 months (5–12 months) survival time is 17.4 months (7–36 months) for these patients (Table 5). No patients in this group had grade 1 disease. The average time to progression and survival in 11 patients with grade 2 disease were 9.18 and 20 months, respectively. The corresponding values for 12 patients with grade 3 disease were 7.75 and 14.8 months (Table 5). No significant differences between the gleason scores were detected ($p < 0.5$). There were 11 with less than 6 and 12 had more than 6 bone metastases, and their average time to progression and survival were 8.9 and 7.9 months, and 19 and 15.9 months, respectively. Again, no significant differences were detected ($p < 0.5$) (Table 5).

There was a total of 28 patients with primary hormone resistance in group 4. In these patients, there was a minimal decrease or no decrease in PSA levels. Chemotherapy was instituted to these patients after 3–6 months and onwards. There were 2 patients with grade 1, 16 with grade 2, and 10 with grade 3 disease. Overall, the average survival was 9.4 months (Table 6). No statistical differences were noted between the gleason scores with respect to survival ($p < 0.05$). Eight patients had less than 6, and 20 had more than 6 bone metastases, again with no statistical difference with respect to survival ($p < 0.05$).

As can be seen from Table 6, there was a significant difference between group 1 and group 2 with respect to average time to progression and survival ($p < 0.02$). The average time to progression and survival were markedly ($p < 0.001$) longer in group 1 and 2 compared to group 3 whose PSA concentrations did not normalise.

**TABLE 5**
**The Patient Characteristics in Group 3**

| Characteristic | No. of Patients | Progression Time (Months) | Survival Time (Months) |
|---|---|---|---|
| Grade | | | |
| 1 | — | — | — |
| 2 | 11 | 9.18 | 20 |
| 3 | 12 | 7.75 | 14.8 |
| No. of bone metastases | | | |
| <6 | 11 | 8.9 | 19 |
| 6 or >6 | 12 | 7.9 | 15.9 |
| Total | 23 | 8.45 | 17.4 |

**TABLE 6**
**The Patient Characteristics in Group 4**

| Characteristic | No. of Patients | Progression Time (Months) | Survival Time (Months) |
|---|---|---|---|
| Grade | | | |
| 1 | 2 | — | 10.5 |
| 2 | 16 | — | 9.2 |
| 3 | 10 | — | 8.5 |
| No. of bone metastases | | | |
| <6 | 8 | — | 8.8 |
| 6 or >6 | 20 | — | 10.8 |
| Total | 28 | — | 9.4 |

## DISCUSSION

Since PSA reproduction and secretion in prostatic epithelial cells are dependent on androgens, androgen blockage can suppress the reproduction and secretion of PSA in both benign and malign prostate tissues[3]. However, prostate cancer cells constitute a heterogeneous group of cells with variable levels of dependency on androgen, leading to observed differences between patients with regard to suppression of PSA levels and the lowest PSA levels achieved. Therefore, it is reasonable to assume that tumours largely consisting of androgen-sensitive cells are more likely to respond to hormonal therapy than those without androgen dependency. Consequently, time to suppression and the lowest concentration of PSA have been proposed as prognostic factors that can be utilised in the monitorisation of the disease.

Following antiandrogen therapy, in approximately 80% of the patients, the PSA concentrations decrease by 80% in the first month[4]. In large patient series, some investigators showed that following hormone therapy, PSA concentrations became normalised in 95% of cases within 3–6 months[5]. Another author found that the response rates were higher and survival was improved in patients in whom PSA levels were normalised within the first 6 months of therapy[6]. PSA has been reported to be a reliable prognostic factor for stable disease in patients with a decrease in PSA concentrations greater than 50% in the first month, and in patients with normal PSA levels in the first 6 months of therapy[7]. Furthermore, the average survival was 42 months in patients in whom the lowest PSA level was within the normal range following hormone therapy compared to those with lowest levels not in the normal range[8]. In another study with 78 patients, the lowest level of PSA obtained 3–6 months after therapy and the time to increase in PSA concentrations had prognostic value. In a study, 5-year survival was 67% in those with normalisation in PSA levels vs. 0% in those without it[10]. Among patients receiving maximum androgen blockage, continued treatment was recommended for those with normalisation within 3–6 months, and in patients with inadequate decrease or no change in PSA concentrations, other alternatives such as chemotherapy were advocated at an early stage based on poor prognostic implications of such levels[11].

Our results are in line with literature data. In our study, the average time to progression in patients with normalisation of PSA levels in the first 6 months of therapy (20.1 months) was significantly longer compared to those without such normalisation (8.45 months) ($p < 0.001$). Again consistent with literature data, there was a significant difference with respect to the time to progression between patients with normalisation at 1–3 months (23.3 months) and those with normalisation at 3–6 months (16.9 months) ($p < 0.02$).

In many studies, gleason score and the number of bone metastases (as pretreatment prognostic factors) have been evaluated in conjunction with the time to decrease in PSA concentrations. Oosterlinck et al.[12] have reported that normalisation of PSA with treatment, the stage of the disease, gleason scores, and performance and pain scores are important prognostic factors, and that the time to progression is significantly shorter in patients without normalisation in PSA levels 3–6 months after treatment and in patients with higher tumour grades. On the other hand, Arai[4] has found no relationship between the decrease in PSA levels after 1 month of therapy and the gleason scores. In the present study, the time to progression in low-risk patients with normalisation of PSA after 1–3 months, grade 1, and less than six bone metastases was significantly longer compared to all other groups. However, among patients with grade 2 and 3, the longer time to progression in group 1 (21 months) compared to group 2 (13.4 months) ($p < 0.01$) suggest that the decrease in PSA is more important from a prognostic standpoint compared to gleason score. Our results suggests that the patients with higher gleason scores should be monitored more closely even if their PSA levels returned to normal at a later stage of treatment.

It has been noted that only in patients with a decrease in PSA concentrations 1–3 months after the start of the therapy, lower number of bone metastases were associated with a prolonged time to progression ($p < 0.01$) when the number of bone metastases and the decrease in PSA were considered jointly. Absence of a similar association in other groups suggest that the number of bone metastases alone do not have a prognostic value, and that it should be evaluated together with other prognostic factors.

In conclusion, normalisation of PSA levels with antiandrogen treatment has significant prognostic implications, as reflected in literature data. Similarly, PSA levels in conjunction with gleason score may provide more valuable information regarding the response to treatment. Thus, the PSA levels can be used as a prognostic factor during the treatment and regular assessment of PSA levels may help to determine the success of treatment or the need for another treatment strategy at an early stage.

**TABLE 7**
**The Average Time to Progression and Survival Data, and the Statistical Comparisons Between the Groups**

| Characteristic | No. of Patients | Progression Time (Months) | Survival Time (Months) | Statistical Significance |
|---|---|---|---|---|
| PSA normalised at month 1–3 | 77 | 23.3 (8–60) | 37.3 (12–96) | |
| PSA normalised at month 3–6 | 21 | 16.9 (8–36) | 31.3 (12–84) | $0.05 < p < 0.02$ |
| PSA normalised at month 6 | 23 | 8.45 (6–12) | 17.4 (10–36) | $p < 0.001$ |
| Hormone-resistant | 28 | — | 9.4 (4–16) | |
| Total | 149 | | | |
